# *Notes from the Field*: A Multipartner Response to Prevent a Binational Rabies Outbreak — Anse-à-Pitre, Haiti, 2019

**DOI:** 10.15585/mmwr.mm6832a6

**Published:** 2019-08-16

**Authors:** Joane Adrien, Yvenel Georges, Pierre D. Augustin, Benjamin Monroe, Andrew D. Gibson, Natael Fenelon, Ludder Fleurinord, Kelly Crowdis, Anna Mandra, Haim C. Joseph, Melissa D. Etheart, Ryan M. Wallace, Haiti-Rabies Field Response Team, Haiti-Rabies Field Response Team, Jesse Blanton, Julie Cleaton, Rene Edgar Condori, Brett Petersen, Yasmeen Ross, David Moran, Joanne Tataryn, Luke Gamble, Fred Lohr, Alasdair King

**Affiliations:** ^1^Field Epidemiology Training Program, Department of Epidemiology and Laboratory Resources, Ministry of Sanitation and Public Health, Port-au-Prince, Haiti; ^2^Ministry of Agriculture, Rural Development, and Natural Resources, Port-au-Prince, Haiti; ^3^Division of High Consequence Pathogens, National Center for Emerging and Zoonotic Infectious Disease, CDC; ^4^Mission Rabies, United Kingdom; ^5^Pan American Health Organization, Port-au-Prince, Haiti; ^6^Christian Veterinary Mission, Port-au-Prince, Haiti; ^7^CDC Haiti.; CDC; CDC; CDC; CDC; CDC; University of the Valley, Guatemala; Public Health Canada; Mission Rabies; Mission Rabies; Merck Animal Health.

Sustained investments in dog rabies vaccination programs and increased access to postexposure prophylaxis have led to a substantial decrease in rabies deaths associated with dogs in the Western Hemisphere (*1*). Despite recent dog vaccination campaigns in Pedernales, Dominican Republic, three human rabies deaths associated with dogs were reported during July–December 2018 in Pedernales, which shares a border with Anse-à-Pitre, Haiti (*2*). Canine rabies is endemic in Haiti and the Dominican Republic; over the past decade, Haiti has reported an eighteenfold increase in laboratory-confirmed canine rabies cases after implementation of an active rabies surveillance program, although none were reported from Anse-à-Pitre (*3*). Haiti conducted a three-phase national dog rabies vaccination campaign during 2017–2018, with the last round occurring during October 16, 2017–May 22, 2018, in the southern third of the country. However, the campaign did not reach the southeastern community of Anse-à-Pitre because of difficult terrain and funding constraints. Although no human or animal rabies cases had been reported from Anse-à-Pitre, health experts from Haiti and Dominican Republic were concerned that dogs from this community could be part of a cross-border enzootic rabies transmission cycle. At the invitation of the Haiti Ministry of Agriculture, a multiagency team deployed to Haiti to vaccinate dogs, conduct human and animal rabies case surveillance, collect retrospective animal and human rabies exposure and case detection data, and evaluate border crossings by dogs. Because it was an emergency outbreak response, CDC determined the activities to be nonresearch.

During January 23–26, 2019, the emergency response team vaccinated 1,331 dogs in the Haitian communities directly adjacent to Pedernales (primarily comprising Anse-à-Pitre and a few surrounding communities). Dogs were marked with temporary, nontoxic paint and a paper collar at the time of vaccination. A mobile phone application was used to geospatially record all vaccinations and conduct postvaccination dog-counting surveys to ensure that target coverage (>70% of susceptible dogs) was achieved (*4*,*5*). Postvaccination surveys identified vaccination marks on 191 (87%) of 220 free-roaming dogs, and enumeration of survey data resulted in an estimated population of 1,750 total dogs in the community (76% vaccination coverage among the total dog population) (Figure).

**FIGURE:**
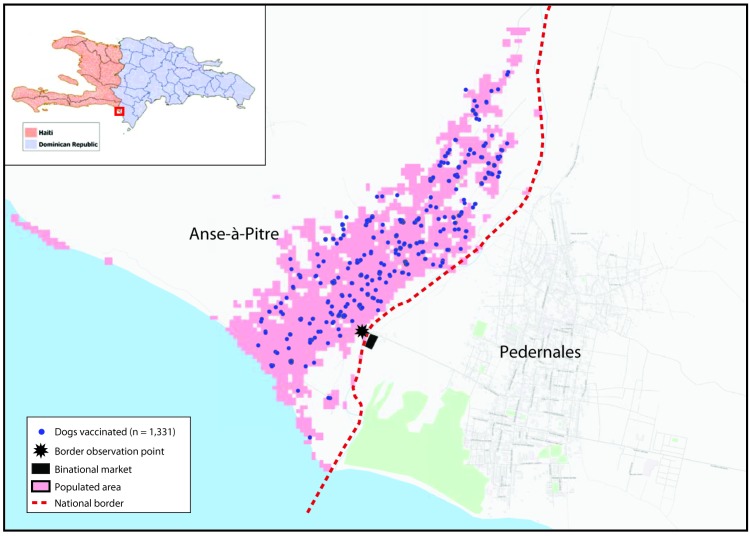
Locations of dogs* vaccinated with rabies vaccine in the border towns of Anse-à-Pitre, Haiti, and Pedernales, Dominican Republic, and of the observation point used during the rabies response investigation and binational market — Anse-à-Pitre, Haiti, 2019 * Determined using global position system.

To identify unrecognized human and animal rabies deaths, a survey of 92 randomly selected households was conducted, and community leaders were consulted. Thirteen dogs with rabies-compatible signs* were identified during May 2018 (one), August (one), November (two), December (two), and January 2019 (seven), suggesting that dog rabies activity increased in November and continued during the January emergency vaccination campaign. In Haitian communities that have implemented the national rabies surveillance program, 50% of dogs that are tested after developing these rabies-compatible signs are confirmed rabid (*3*). Household surveys found no suspected human rabies deaths in the preceding 12 months in Anse-à-Pitre. Household surveys identified 11 persons who had been bitten by dogs in the past year, only two of whom had sought medical evaluation (22%). None reported receiving rabies vaccination, and all were healthy at the time of survey. Four additional persons who had been bitten by dogs were identified during response activities. All 15 exposed persons identified during response efforts who had not initiated the rabies vaccination series were referred to the Anse-à-Pitre government hospital.

Training on medical management and reporting of human rabies cases and dog bite events was conducted at the Anse-à-Pitre government hospital. The response team provided the hospital with 100 doses of human rabies vaccine and 500 rabies prevention information comic books. Human rabies immune globulin, a World Health Organization–recommended component of the rabies postexposure prophylactic treatment regimen, is not routinely available in Haiti and was not available in the Anse-à-Pitre government hospital.

Three surveillance officers were trained to conduct rabies field investigations in Anse-à-Pitre using a custom-built mobile device application to investigate and report rabies exposures and manage suspected rabid animals. This application is used by Haiti’s national animal rabies surveillance program, but had not been implemented in Anse-à-Pitre and the surrounding communities until the emergency response. Surveillance officers collected brain tissue from two dogs with suspected rabies, one of which was found dead by the owner and a second that died a day after being quarantined. Both specimens tested negative for rabies by direct fluorescent antibody testing at the National Veterinary Laboratory, Haiti. From the start of the response until July 30, 2019, surveillance staff members investigated 26 biting dogs in the Anse-à-Pitre community; 17 (65%) of the dogs were known to have been vaccinated during the campaign, and none had signs consistent with rabies virus infection.

Observers recorded six dogs crossing from Pedernales into Anse-à-Pitre with their owners during a 12-hour period. During the same period, a field survey in the Pedernales binational market identified 14 free-roaming dogs, one of which had Haiti’s vaccination mark.

On January 24, 2019, a binational rabies meeting^†^ was held in Anse-à-Pitre. General consensus was obtained on the importance of coordinated binational canine rabies vaccination and surveillance efforts, and participants affirmed their interest in pursuing binational rabies prevention measures.

Dog bites and suspected canine rabies cases are underdetected in Anse-à-Pitre. Intermittent canine rabies cases have likely occurred during the past year; however, a potential rise in cases began in November 2018, 5 months after the first human death in Pedernales. The most cost-effective way to prevent human rabies deaths in Anse-à-Pitre and Pedernales is through annual coordinated cross-border dog vaccination campaigns until canine rabies elimination is achieved. The emergency response was successful in achieving vaccination targets and highlighting this important binational public health issue. As both natural and human-associated binational dog movements were confirmed during this investigation, collaborative interventions should be pursued to eliminate canine rabies from border communities. Continued surveillance will be necessary to assess the effectiveness of the interventions.

## Footnotes

* Acute development of aggression, hypersalivation, or behavioral changes preceding the dog’s death. Unpublished data from Haiti’s national rabies surveillance program has found that approximately 50% of dogs with these clinical signs, when available for testing, are laboratory-confirmed to be infected with rabies virus.

† Attendees included representatives from the Ministry of Agriculture and Health, Haiti; Ministry of Health, Anse-à-Pitre, Haiti; CDC; Pan American Health Organization, Port-au-Prince, Haiti; and the Pedernales Public Health Department, Dominican Republic.

## References

[1] Freire de Carvalho M, Vigilato MAN, Pompei JA, Rabies in the Americas: 1998–2014. PLoS Negl Trop Dis 2018;12:e0006271. 10.1371/journal.pntd.0006271 29558465 PMC5877887

[2] Mandra A, David Morán D, Santana PV, Notes from the field: rabies outbreak investigation—Pedernales, Dominican Republic, 2019. MMWR Morb Mortal Wkly Rep 2019;68:704–6. 31415487 10.15585/mmwr.mm6832a5 PMC6818700

[3] Wallace RM, Reses H, Franka R, Establishment of a canine rabies burden in Haiti through the implementation of a novel surveillance program [corrected]. PLoS Negl Trop Dis 2015;9:e0004245. 10.1371/journal.pntd.0004245 26600437 PMC4657989

[4] World Health Organization. WHO expert consultation on rabies. Third report. WHO technical report series, no. 1012. Geneva, Switzerland: World Health Organization; 2018. https://www.who.int/rabies/resources/who_trs_1012/en/

[5] Gibson AD, Mazeri S, Lohr F, One million dog vaccinations recorded on mHealth innovation used to direct teams in numerous rabies control campaigns. PLoS One 2018;13:e0200942. 10.1371/journal.pone.0200942 30048469 PMC6062050

